# The role and application of bioinformatics techniques and tools in drug discovery

**DOI:** 10.3389/fphar.2025.1547131

**Published:** 2025-02-13

**Authors:** Shujun Zhang, Kaijie Liu, Yafeng Liu, Xinjun Hu, Xinyu Gu

**Affiliations:** ^1^ Department of Infectious Diseases, The First Affiliated Hospital, College of Clinical Medicine, Henan University of Science and Technology, Luoyang, Henan, China; ^2^ Henan Medical Key Laboratory of Gastrointestinal Microecology and Hepatology, Luoyang, China; ^3^ Department of Oncology, The First Affiliated Hospital, College of Clinical Medicine, Henan University of Science and Technology, Luoyang, Henan, China

**Keywords:** bioinformatics, omics, drug discovery, precision medicine, targeted therapy

## Abstract

The process of drug discovery and development is both lengthy and intricate, demanding a substantial investment of time and financial resources. Bioinformatics techniques and tools can not only accelerate the identification of drug targets and the screening and refinement of drug candidates, but also facilitate the characterization of side effects and the prediction of drug resistance. High-throughput data from genomics, transcriptomics, proteomics, and metabolomics make significant contributions to mechanics-based drug discovery and drug reuse. This paper summarizes bioinformatics technologies and tools in drug research and development and their roles and applications in drug research and development, aiming to provide references for the development of new drugs and the realization of precision medicine.

## 1 Introduction

Drug development begins with the discovery of a disease and its changes. When a disease threatens human health and reduces the quality of life, drugs are created. The ideal drug should not only reduce symptoms and treat the disease, but also have a high safety profile, few side effects, and low research costs. However, this is undoubtedly a huge challenge. The process of drug development is both lengthy and intricate, demanding a substantial investment of time and financial resources. Bioinformatics shows great potential in drug discovery. Bioinformatics is an interdisciplinary science, which uses computer science, information technology, applied mathematics and statistics methods to gather, process, store, disseminate, analyze and interpret biological information in life science research. In May 1985, American scientist Robert Sinsheimer first proposed the Human Genome Project, which is expected to determine the entire DNA sequence of the human genome within 15 years, decode about 25,000 genes in the human body, and map the human genome. After unremitting efforts, finally in April 2003, the Human Genome Project was officially completed, and the composition of the human genome was determined: 46 chromosomes, 3 billion bases and 30,000 protein-coding genes. The completion of this project marked the beginning of the rapid development of bioinformatics.

The origins of modern drug development can be traced back to the observation that certain natural substances of plant or animal origin are beneficial to human health and can be used to treat diseases (Pina et al., 2009). Advances in organic chemistry have made it possible to extract active molecules, while compounds with similar properties have been synthesized from their structural knowledge. With the synthesis of a large number of compounds and their testing for biological activity in laboratory models of human diseases based on cells, tissues, and organs, “phenotypic screening” is increasingly becoming an important means of drug development (Moffat et al., 2017). Bioinformatics provides a new research direction and innovative method for drug development. For example, in cancer research, bioinformatics can analyze large-scale cancer genomic data to discover new mechanisms of tumorgenesis, novel targets, and potential drugs. Bioinformatic analysis can expedite the identification of drug targets, enhance the screening and optimization of drug candidates, and facilitate the characterization of side effects and prediction of drug resistance. High-throughput data, such as genomic, transcriptomic, proteomic, and metabolomic data, make important contributions to mechanics-based drug discovery and drug reuse. By analyzing large amounts of biological data, researchers can better understand the pathogenesis of diseases and discover diagnostic markers and therapeutic targets, thereby supporting the development of personalized and precision medicine. With the increasing amount of biological data and the advancement of computing technology, the position of bioinformatics in medical research will be further enhanced.

In this review, we summarized the bioinformatics technologies and tools in drug development and their roles and applications in drug development, providing references for disease treatment and the development of new drugs.

## 2 Bioinformatics in drug discovery

### 2.1 Biological database

Biological data is the basis of bioinformatics technology, including genome data, protein sequence data, gene expression data and biomarker data. These data are derived from biological experiments and research, and their collection, storage, management and analysis constitute the key links of bioinformatics technology. As a result, many biological databases have emerged to store, manage and share biological data, while integrating existing resources such as research results and technical information. Table 1 shows some commonly used biological databases that enable researchers to search for information about biological research to facilitate the development of new drugs.

**TABLE 1:** Commonly used biological database in drug discovery.

Type	Database	Content	References
Genomic database	NCBI RefSeq NCBI GenBank EMBL DDBJ	store genome sequence data	O'Leary et al. (2016), Sayers et al. (2022), Thakur et al. (2024), Fukuda et al. (2021)
Protein sequence database	UniProtKB/Swiss-Prot TrEMBL UniParc UniRef World-2DPAGE wwPDB	store protein sequence data	Boutet et al. (2007), Kriventseva et al. (2003), Leinonen et al. (2004), Suzek et al. (2007), Hoogland et al. (2008), Behzadi and Gajdács (2022)
Gene expression database	NCBIGEO ArrayExpress	store gene expression chip data	Bartha and Győrffy (2021), Sarkans et al. (2021)
Biomarker database	Human Metabolome Database KEGG BioCyc ChEMBL	store biomarker data	Kanehisa et al. (2023), Paley and Karp (2021), Wishart et al. (2022), Karp et al. (2019)

Over the past decade, bioinformatics tools, such as computer methods and high-throughput screening techniques, have played an important role in accelerating drug discovery. These methods effectively support the screening and development of natural, synthetic and semi-synthetic compounds, and provide an important boost for the research of potent drugs or lead molecules. In-depth research into natural products and their derivatives has successfully contributed to about 34% of newly approved drugs (Patil and Masand, 2021). As one of the major diseases threatening human health, the precision treatment of tumor has been broken through due to the development of targeted therapy. Given the complexity of cancer pathogenesis and the difficulty of anticancer drug development, we need to expand chemical and biological resources to provide more potential molecular scaffolds for anticancer drug discovery and development. The establishment of databases can efficiently manage and analyze relevant data information, such as SuperNatural (Dunkel et al., 2006), NPACT (Rosita and Begum, 2020), TCMSP (Ru et al., 2014), CancerHSP (Tao et al., 2015), TCMID (Xue et al., 2013) and Phytochemica (Pathania et al., 2015), etc. It covers multi-dimensional information such as chemical structure, physical and chemical properties, target protein interaction, distribution, absorption, metabolism, excretion, toxicity and biological activity. Table 2 summarizes databases of value for cancer drug development that have included more than 100,000 anticancer compounds, although the information may be incomplete. In addition, databases have been found to be useful for identifying lead compounds against pharmacological targets (Barlow et al., 2012). The information provided by the database not only facilitates drug discovery, but can also be used to generate computational models such as quantitative structure-activity relationships (QSAR), pharmacophore models, and protein-ligand interactions (docking studies) to further screen for biologically active natural and synthetic molecules (Tung, 2014).

**TABLE 2:** Some databases of value for anticancer drug discovery.

Name	Website	Role	References
CancerResource	http://data-analysis.charite.de/care/	(1) Provides cancer-related drug-target relationships, genomics (mRNA, non-synonymous mutations), cellular fingerprints, mutation data, and drug sensitivity information (2) Regularly updates on tumor heterogeneity, tumor response to anticancer therapy, and tumor stratification	Gohlke et al. (2016)
CancerHSP	http://lsp.nwsuaf.edu.cn/CancerHSP.php	(1) Consists of six parts: herbal medicine, herbal ingredients, target of action mode, biological activity for different cell lines, primary site of cell lines and pharmacokinetic properties (2) Evaltates and studies protein targets for each compound at the molecular level	Tao et al. (2015)
canSAR	http://cansar.icr.ac.uk/	(1) The world’s largest public database of pharmacability evaluation and cancer drug discovery for identifying and validating targets (2) Provides detailed information on chemical probes, biological activity, target engagement biomarkers, and drug combinations	Micco et al. (2023)
NPACT	http://crdd.osdd.net/raghava/npact/	(1) Provides information on the anti-cancer characteristics evaluated *in vivo* and *in vitro* experiments of various cancer cell lines (2) Provides information on protein targets and drug receptor/target interactions	Mangal et al. (2013)
NPCARE	http://crdd.osdd.net/raghava/npact/	A database of about 6,500 unique natural compounds and 2,566 isolated extracts collected from literature and online resources	Choi et al. (2017)
PharmacoDB	https://pharmacodb.pmgenomics.ca/	Provides information on cancer data sets, tissues, cell lines, compounds, and genes	Smirnov et al. (2018)

### 2.2 Molecular docking computing tools in drug discovery

Drug discovery is a formidable endeavor, with the identification of the optimal lead compound being a critical determinant of a project’s success. In 2016, the Tufts Center for the Study of Drug Development noted that while the average time for drugs to enter clinical trials has decreased over the past decade, the success rate of winning approval from the U.S. Food and Drug Administration (FDA) has declined (DiMasi et al., 2016). Scientific advances have changed the way new bioactive molecules are generated in drug research. Computer aided drug design (CADD) can more quickly guide experimental studies to find the best compounds, helping to reduce the cost and time of drug discovery. In CADD, techniques like molecular docking and virtual screening (VS) serve as invaluable complements to the resource-intensive and costly high-throughput screening (HTS) experimental process.

**FIGURE 1:**
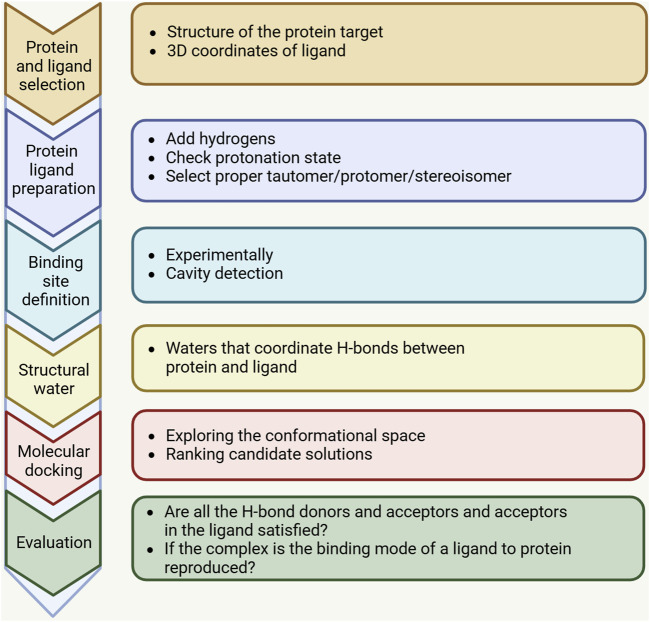
A standard docking workflow demonstrates the essential steps shared by all docking protocols. First, the three-dimensional structures of the target macromolecule and small molecule need to be selected and prepared in accordance with the chosen docking method. The binding site, identified through computational tools or experimental data, may incorporate water molecules or structured water. After docking is completed, the results should be analyzed, and the highest-scoring binding modes should be selected and evaluated.

Advances in computational technology and parallel hardware have enabled computer methods, particularly structure-based drug design (SBDD) methods, to accelerate the selection of new targets by identifying hit points in the drug discovery process, thereby optimizing the screening of lead compounds. Molecular docking is a widely employed computational, structure-based method in drug design, extensively used since the early 1980s, and its main goal is to achieve molecular recognition by predicting binding patterns and affinity (Kuntz et al., 1982). Significant improvements in computer performance and the abundance and ease of use of small molecule and protein structure data have promoted the wide application of molecular docking technology. Initially, molecular docking was mainly used for binding between small molecules and target proteins, but in the last decade, molecular docking techniques have expanded to protein-protein docking, nucleic acid (DNA and RNA) -ligand docking, and nucleic acid-protein-ligand complex docking studies. The molecular docking process usually consists of two key steps: predicting the conformation of the ligand and its orientation and orientation at the protein binding site (i.e., pose), and evaluating the pose quality using a scoring function. Figure 1 shows the key steps of the molecular docking process. The docking process requires the active compound to have a higher score than the known inactive compound. However, achieving this level of accuracy is challenging and is frequently influenced by numerous factors in the protein’s external environment. Therefore, the current docking algorithms are mainly concerned with correctly predicting the pose of ligands and evaluating the quality of pose.

Fragment-based screening is designed to identify small chemical fragments that exhibit weak binding affinity to the target protein, thereby helping to determine the point of interaction where the protein binds to the ligand. This approach allows for more efficient sampling of chemical Spaces than using larger, more complex molecules, allowing for a broader and more diverse exploration of chemical Spaces. In addition, small fragments typically do not contain interfering groups and are therefore less likely to impede favorable ligand-protein interactions so that the optimal binding site is not obscured by non-binding elements. In theory, virtual fragment screening using molecular docking is feasible, but smaller virtual fragments present significant challenges to the docking process. Because fragments form fewer critical interactions with binding sites, resulting in low docking scores, potential fragment hits may be missed if they interact weakly with proteins. The disparity in free energy between various binding modes of fragments is significantly less than that between larger compounds, thereby making it increasingly challenging to differentiate the correct binding modes from the incorrect ones due to the inherent inaccuracies in the current scoring function. In addition, due to the relatively small size of the fragments, their docking postures may be mixed, and the fragments may bind to multiple subregions and exhibit similar physico-chemical properties within a binding pocket. In such instances, interpreting docking results proves challenging, and post-processing resuits can be quite time-consuming (Bian and Xie, 2018).

The applications of molecular docking in drug discovery are extensive, including structure-activity studies, virtual screening of potential lead compounds, lead optimization, provision of hypotheses in combination to facilitate mutation prediction, and support in X-ray and cryo-EM crystallography to align substrates and inhibitors with electron density (Stanzione et al., 2021). Computational screening has successfully identified highly concentrated subpopulations of potentially active compounds by identifying large libraries of compounds that are similar to known inhibitors or complementary to target structures, from which their activity can then be further experimentally verified (Ripphausen et al., 2010). Molecular docking can predict the optimal location, orientation and conformation of drug candidates when they bind to proteins, thus providing effective support for future lead optimization (Joseph-McCarthy et al., 2007). A precise comprehension of ligand binding sites and mechanisms aids in the rational design of structural modifications to enhance protein-ligand interactions, boost activity, and prevent alterations that may cause conflicts between proteins and ligands.Molecular docking has achieved remarkable success in structure-based drug design (SBDD), where several marketed drugs, such as Nelfinavir (Kaldor et al., 1997), zanamivir (von Itzstein et al., 1993), imatinib (Druker and Lydon, 2000), and Eldafitinib (Squires et al., 2011), as well as several clinical drug candidates, have been discovered or optimized with the help of computational methods (Talele et al., 2010). Although docking technology is at a mature stage, there is still a lot of room for improvement. At present, the application of molecular docking computing tools in drug discovery faces the problem of lack of suitable scoring function and search algorithm, which is the main shortcoming of current docking technology.

### 2.3 Omics in drug development

The process of drug discovery and development is both lengthy and intricate, demanding substantial investments of time and financial resources. Against the backdrop of declining productivity, the rapid increase in drug development costs could adversely affect the sustainability of the pharmaceutical industry. There are many factors that influence drug discovery and development, Includes Medicinal Objective, The Ability of Medicinal Chemists, Screening Facilities, Drug Development Facility and Cost of New Drugs, etc., (Sharma et al., 2023). Traditional drug discovery processes are cumbersome, expensive and time-consuming. The pharmaceutical industry faces unprecedented challenges, especially a general shortage of late-stage R&D channels. Reversing current trends will necessitate a multifaceted strategy, including rigorous and dependable target selection and validation, enhanced animal model systems, and the identification of reliable biomarkers and alternative endpoints. Reversing current trends will necessitate a multifaceted strategy, including rigorous and dependable target selection and validation, enhanced animal model systems, and the identification of reliable biomarkers and alternative endpoints. The advent of the era of omics application provides a powerful technical resource for further understanding of disease complexity and drug development. With the advent of multi-omics technologies, encompassing genomics, transcriptomics, proteomics, and metabolomics, our comprehension of diseases continues to expand profoundly. These multi-omics technologies build a progressive analysis framework from genetic basis to environmental exposure effects, in-depth analysis of disease pathogenesis, pathophysiological processes and molecular basis, and provide strong support for scientific formulation of precision treatment strategies.

#### 2.3.1 Genomics in drug discovery

As the support of the basic structure and function of life, genes carry the complete information of the whole life process, including race, blood type, pregnancy, growth and apoptosis. Human genome data plays an important role in drug research and development, which is reflected in the identification and validation of drug targets, the effectiveness and specificity evaluation of the combination of compounds and targets, and the selection of clinical trial endpoints. The successful completion of the Human Genome Project in 2003 triggered a revolutionary progress in the field of biotechnology and gave birth to the rapid development of “omics,” a comprehensive and diverse discipline. Breakthroughs in high-throughput sequencing technology have led to the successful identification of thousands of genes, while the significant decline in sequencing costs has pushed the scope of research beyond the human genome to a wider range of areas, making comparative genomics an important tool for identifying pathogen-specific targets. The proliferation of genome sequencing technology has made it easier for researchers to access and apply this technology. With these advances, it has come to be understood that the proteins encoded by the genome are not only the main proximal effector molecules in biology, but also the core of drug targets (Finan et al., 2017).

By examining DNA sequences and deciphering the genetic information in the genome, genomics enables scientists to precisely identify specific genetic mutations and identify potential drug targets to develop precise targeted therapies. The maturity of genomics technologies indicates that the data generated by system integration will significantly accelerate the drug discovery and development process. Genomic research has profoundly enhanced our comprehension of disease biology and diagnostic practices. For example, the discovery of Cathepsin K as a molecular target in osteoporosis and the sequencing of all members of the gene superfamily (e.g., G protein-coupled receptors, ion channels, nuclear hormone receptors, proteases, kinases, etc.) has important implications for drug discovery (Lappano and Maggiolini, 2011). The availability of large amounts of genomic sequence information on pathogenic and non-pathogenic bacteria has made it possible to detoxify mechanisms and develop treatments that specifically target the metabolic pathways of pathogens (Land et al., 2015). In addition, genomics helps us understand the effects of drugs. For example, the spider venom protein PcFK1 impedes the proliferation of Plasmodium falciparum, though the underlying mechanism remains elusive. Through sequence analysis, it was found that PcFK1 was homologous to the substrate sequence of PfSUB1 protein of Plasmodium falciparum enzyme, and it was speculated that PcFK1 played an antimalarial role by inhibiting PfSUB1. Further docking predictions and *in vitro* experiments confirmed the hypothesis that PfSUB1 could be used as a drug target (Bastianelli et al., 2011). Genomic analysis can also help change the use of existing drugs. Galactosyl urea sugar (Galf) is a crucial element on the cell surface of numerous bacterial pathogens, and its synthesis depends on UDP-galactopyranose mutase (UGM) (Kincaid et al., 2015; Gruber et al., 2009). Due to the lack of UGM in the human body, UGM has been used as an ideal drug target (Pedersen and Turco, 2003). UGM encoded by the GLF gene was later found in eukaryotic single-cell pathogens and nematodes. Due to the significant difference between eukaryotic UGM and prokaryotic UGM, drugs developed against bacterial pathogens cannot be used against eukaryotic pathogens, leading to difficulties in drug reuse. Nevertheless, should an efficacious drug be formulated against a specific eukaryotic UGM through genomic analysis, it is highly probable that this drug could be repurposed to combat another eukaryotic pathogen.

In the targeted drug development process, bioinformatics approaches typically involve three basic steps. First, key genes in the pathogen need to be identified as potential drug targets. Second, check whether there are homologous genes of these genes in the host; Finally, drugs should target specific pathogens to minimize the development of resistance. Through the analysis of large amounts of genomic data, bioinformatics can help researchers identify genes associated with the development of diseases and develop drugs that target these genes. In addition, the development of personalized treatment plans based on individual genomic information will help promote the development of precision medicine.

#### 2.3.2 Transcriptomics in drug discovery

RNA interference can regulate a variety of cellular processes, and RNA has been regarded as a novel drug. Thus, digging deeper into transcriptomic data may reveal more RNA molecules with potential drug or drug target functions (Xia, 2017). Using bioinformatics tools, functionally important Rnas can be identified in millions of different transcripts. Any RNA fragment with an important function can be a potential drug target. Bioinformatics methods are used to analyze all the collections of RNA in a particular cell or tissue under specific conditions, known as transcriptome data, which can help identify changes in the expression of disease-related genes, thereby providing potential biomarkers for diagnosis and treatment of diseases. Transcriptomic data are increasingly employed to identify differentially regulated genes, alternative splicing isoforms, and divergent transcription start and end sites between patients and controls (Arvaniti et al., 2016; Mlera et al., 2016). The role of transcriptomic data analysis in drug discovery is primarily twofold: first, it aids in the identification and optimization of drug candidates through phenotypic screening; second, it facilitates the identification of potential drug targets.

With the enhanced capability to synthesize vast quantities of compounds and evaluate their biological activity in laboratory models of human diseases using cells, tissues, and organs, phenotypic screening has emerged as a pivotal approach in drug development (Moffat et al., 2017). Phenotypic screening evaluates the impact of compounds on cells, tissues, or model organisms, identifying effective agents based on their capacity to modify biochemical, physiological, or pathological processes within the model. This approach allows the compound to be further studied as a potential drug even if the molecular mechanism of its action is not fully understood. Phenotypic screening has significant advantages in the identification of active ingredients, and the discovery of artemisinin is a classic application case, which has become one of the most effective drugs against malaria (Miller and Su, 2011). Target-based approaches are generally suitable for drug development for relatively simple diseases such as monogenic inherited diseases, while phenotypic screening has shown better results in drug development for multi-etiological diseases such as polygenic inherited diseases (Swinney, 2013; Swinney and Anthony, 2011). In cancer research, due to the high genetic diversity among tumor cells, phenotypic screening has great potential for application in cancer drug development (Shoemaker, 2006). In addition, phenotypic screening of FDA-approved drugs for drug reuse is cost-effective. For example, this approach has led to the discovery of promising enterovirus inhibitors, anticancer agents, anti-aging agents, and variant Bcr-Abl inhibitors against chronic myeloid leukemia (Ulferts et al., 2016; Ozsvári et al., 2016; Snell et al., 2016; Singh et al., 2017). In the screening process of anticancer drugs, phenotypes are often defined as gene expression profiles or metabolomics profiles. Therefore, two approaches are usually taken when researching anti-cancer drugs: one is to restore abnormally expressed genes in cancer cells to normal levels, and the other aims to eradicate cancer cells by inducing apoptosis (Moffat et al., 2014). The process of phenotypic screening generally involves screening a large number of compounds as drug candidates, monitoring the phenotypic changes of each compound, developing desirable criteria and sequencing compounds, and finally selecting compounds that produce desirable biological effects for further testing and validation (Eder et al., 2014). In phenotypic screening studies utilizing gene expression profiles, bioinformatics can advance drug discovery by developing objective and rational indicators of drug desirability (IDDs). Idds can complement therapeutic metrics based on various pharmacokinetic models to evaluate efficacy and safety at different drug concentrations. Some scholars believe that the lack of clear Idd may be one of the reasons for the low success rate of phenotypic screening methods in drug discovery (Eder et al., 2014).

When the mechanism of action and therapeutic targets are obscure, forecasting drug effects becomes challenging, and it constrains the potential to develop enhanced compounds grounded in the mechanism of action. With the deepening understanding of the molecular mechanism of disease, target-based drug development methods have been widely used. Unlike phenotype-based approaches, target-based approaches begin with the pathogenesis of the disease, and potential drug targets have been shown to be closely related to the disease process. In target-oriented models, drug development begins by identifying proteins associated with disease onset and progression, known as drug targets, which are suitable for the study of small molecule drugs or monoclonal antibodies. The method necessitates experimental validation to establish the causal relationship between the target and the disease. However, a growing number of studies have shown that using this method for drug research is less efficient and less successful. Further analysis shows that the failure of target-based drug development is due to the weak ability of laboratory models to predict the pathogenesis of human diseases. Poor external validity of preclinical human disease models; Isolated cells and tissues may not accurately represent the entire organism, and animal models often fail to simulate human pathophysiological processes well. In addition, there is a high rate of false findings in preclinical science (Hingorani et al., 2019). It has been estimated that using genome-wide association studies as the primary source of information for drug target identification, replacing traditional preclinical studies, is expected to reduce the risk of later failure. Through large-scale genetic studies, combined with genomics and electronic health record data in the healthcare system, it is possible to significantly improve the resolution of disease endpoints, which may radically improve the success rate of drug development.

#### 2.3.3 Proteomics in drug discovery

Proteins are essential molecules in almost all living things. They provide scaffolds for cells and play key roles in metabolism, biological signaling, gene regulation, protein synthesis, and solute transport across membranes. The abnormal regulation of protein function is one of the important factors in the pathology of many diseases. Therefore, understanding how the proteome is disturbed by disease is a central goal of biomedical research. Bioinformatics tools are able to predict the three-dimensional structure of proteins, which is critical for understanding their function and drug design. In addition, bioinformatics can also help explain the function of proteins, providing important clues to study their role in disease.

The proteins encoded by the genome are not only the main effector molecules in biology, but also constitute the main class of drug targets. Almost all small molecule drugs and biological therapies work by disrupting the function of proteins. Therefore, drug development is based on the identification of proteins or targets associated with the disease. Once the targets are identified, specific compounds for those targets need to be developed. Because a transcribed gene may or may not be translated differentially (Xia et al., 2011; Xia, 2015; Gilbert et al., 2007), and because different proteins degrade at different rates, transcriptomic data are generally not good predictors of protein abundance (Swinney and Anthony, 2011, Hughes et al., 2011). Conversely, the characterization and comparison of proteomic data between patients and controls often prove more efficacious in identifying potential drug targets. Recently, Cifani et al. (2018), Kwok et al. (2023) proposed ProteomeGenerator, a hybrid proteomic framework. The method used sample specific control to calibrate the matching results of target decoy database, and significantly improved the accuracy of isomer identification in atypical proteome.

The bioinformatics tools used for proteomic data analysis closely resemble those employed for transcriptomic data analysis, as both facilitate phenotypic screening and drug target discovery through proteomic insights. Traditional methods usually focus on one or a few proteins; However, with the continuous advancement of sample separation technology and mass spectrometry technology, today’s research is able to analyze complex biological systems as a whole. Typical proteomic experimental strategies based on mass spectrometry (MS) can be divided into top-down and bottom-up methods according to the size of the protein. In top-down proteomics, complete protein molecules are analyzed directly by mass spectrometry (Doudna and Charpentier, 2014); In the bottom-up approach, the protein sample is first hydrolyzed and digested into peptides, which are then analyzed in a mass spectrometer (Wang et al., 2014). The swift advancement of proteomics has led to an array of downstream bioinformatics analysis techniques, elucidating the intricate relationship between protein regulatory mechanisms and phenotypic behavior at the molecular level (Renaud et al., 2016). Currently, proteomic data comes from almost all model organisms and is stored in public databases such as PaxDB (Wang et al., 2012). These data have greatly promoted the application and development of indicators such as prediction of translation efficiency (Prabhakaran et al., 2015; Chithambaram et al., 2014).

#### 2.3.4 Metabolomics in drug discovery

Metabolic abnormalities can disrupt metabolic pathways, leading to either the accumulation or depletion of metabolites, which are increasingly recognized as key indicators of disease. Metabolite signatures that are highly correlated with the subject’s phenotypic information dimension can be used to predict disease diagnosis, prognosis, and to monitor treatment. Metabolomics, centered on small molecule metabolites, has emerged as a crucial tool for uncovering the potential mechanisms of various human diseases and exploring therapeutic possibilities. It not only identifies functional biomarkers linked to phenotypic variations but also characterizes alterations in biochemical pathways as early indicators of pathological dysfunction and impairment prior to disease onset. Detecting and identifying changes in small molecule metabolites or metabolic pathways enhances the understanding of disease pathophysiology and aids in identifying therapeutic targets.

Advances in metabolomics technology offer a non-invasive, high-throughput tool, often categorized into targeted and non-targeted analyses, demonstrating significant value in metabolite characterization. This enables researchers to conduct comprehensive analyses of small molecule metabolites via mass spectrometry to gain insights into metabolic functions. Non-targeted metabolomics uncovers extensive unknown metabolic information, while targeted approaches, focusing on specific sets of metabolites, are generally more sensitive and reproducible. Metabolomics, the science of characterizing both known and unknown small molecule metabolites, serves as an ideal tool for disease characterization and monitoring, as well as for studying the pathophysiology and biochemical features of diseases within body systems. The primary methods encompass metabolic phenotyping, metabolic fingerprinting, metabolic analysis, and targeted metabolite analysis. The metabolic phenotype reflects the characteristic alterations in metabolic responses to pathophysiological stimuli at a given moment. Metabolomics, based on small molecule metabolites, offers distinct advantages over other omics approaches. While genomics may have limited influence on the expression of protein functions, metabolomics directly captures biochemical reactions to stimuli (Li et al., 2021; Wozniak et al., 2020; da Silveira et al., 2020). Unlike genomics, transcriptomics, and proteomics, metabolomics provides a dynamic and detailed analysis of metabolic functions within living systems (Keshavan, 2021). As the downstream product of the genome, transcriptome, and proteome, the metabolome encompasses small molecule metabolites linked to specific metabolic phenotypes. The process of metabolomic analysis for small molecules includes experimental design, selection of biological subjects, sample collection and preparation, metabolite extraction, data acquisition and processing, data analysis, and ultimately, deriving insights through biomarker discovery and functional interpretation (Qiu et al., 2023).

Metabolomics provides patients with more precise tools than traditional biomarkers. Technological advancements have unveiled novel opportunities to examine the metabolic aspects of disease. Primary analytical techniques for endogenous molecules encompass nuclear magnetic resonance (NMR) and mass spectrometry. Mass spectrometry is able to identify low-abundance metabolites, while NMR helps reveal metabolic changes in key pathways. Recent studies have concentrated on mapping the spatial distribution of small molecule metabolites, identifying their active constituents, and conducting trend analysis and characterization (Yuan et al., 2021; Fan et al., 2021). Mass spectrometry (MS) has become a pivotal tool in detecting small molecule metabolites, offering a comprehensive framework for understanding metabolic changes, from systemic to single-cell levels. Metabolomics-based mass spectrometry methods enable the rapid discovery of small molecule metabolites, advancing our understanding of metabolic mechanisms in a variety of diseases and enhancing our ability to monitor metabolic changes in clinical Settings. Mass spectrometry, when integrated with liquid chromatography, significantly enhances the versatility and sensitivity of metabolite identification and quantification. This powerful combination facilitates the comprehensive exploration of numerous small molecule metabolites in biological samples, thereby mapping key metabolic alterations associated with disease. Currently, no single analytical method or instrument is capable of identifying the entire metabolome. Some studies suggest maximizing the potential of metabolomics data through joint platforms (Xuan et al., 2020; Garcia-Perez et al., 2020). Recently, scientists have progressively developed a comprehensive metabolic profile to uncover potential mechanisms and metabolic networks for exploring biomedical therapeutic targets.

## 3 Application of bioinformatics in drug development

### 3.1 Bioinformatics and anticancer drug research

The discovery of new drugs is crucial for cancer treatment and precision medicine. Conventional drug discovery methods predominantly depend on *in vivo* animal testing and *in vitro* drug screening, yet these approaches are frequently expensive and challenging. The explosion of omics data over the past decade has opened up new opportunities for cancer drug research, significantly increasing the efficiency of drug discovery. The integration of high-throughput transcriptome data with drug response data has become a cornerstone in biomarker identification and drug efficacy prediction. Furthermore, biological network theories and methodologies have proven effective in anticancer drug discovery, exemplified by studies leveraging protein-protein interaction networks, drug-target networks, and disease-gene networks. One of bioinformatics’ pioneering contributions to drug target identification was the discovery of sequence homology between the anthroposarcoma virus gene and platelet-derived growth factor (PDGF) through straightforward string matching (Waterfield et al., 1983; Doolittle et al., 1983). This discovery makes PDGF a target for cancer drugs (Pietras et al., 2003; Papadopoulos and Lennartsson, 2018; Zou et al., 2022) and leads to two new approaches. Firstly, viral transformation factors may solely function by converting transient growth factor expression into sustained expression, indicating that growth factors are crucial targets for anticancer drug development. Secondly, any factor that modulates gene expression patterns is likely to induce cancer. This novel conceptual framework for cancer biology has facilitated mechanism-based advancements in anticancer drug development in subsequent years.

Small cell lung cancer (SCLC) is an exceptionally aggressive neuroendocrine malignancy characterized by rapid proliferation, extensive metastasis, significant drug resistance, and a poor prognosis (Megyesfalvi et al., 2023). By integrating mRNA, protein, and phosphorylation data from 107 SCLC tumors, Liu et al. employed an unsupervised clustering approach based on non-negative matrix factorization (NMF) to categorize SCLC into four distinct subtypes: NMF1, NMF2, NMF3, and NMF4 (Liu et al., 2024). Multi-omics analysis revealed that the NMF1 subtype was predominantly associated with processes such as the cell cycle, DNA damage repair, chromatin remodeling, and epigenetic regulation, exhibiting a robust response to ATR and TOP1 inhibition. The NMF2 subtype is characterized by the highest level of NOTCH ligand delta-like protein 3 (DLL3) protein, suggesting that this subtype may benefit from DLL3-targeted therapy. Further phosphorylated proteomic analysis revealed that the RTK signaling pathway is significantly upregulated in the NMF3 subtype, so targeting RTK could represent a promising strategy for treating the NMF3 subtype. The NMF4 subtype is distinguished by elevated MYC expression and a predominant enrichment of RNA metabolic pathways, and amplification of the gene AURKA is highly correlated with this subtype, further supporting the potential of AURka-targeted therapies. A multi-omics analysis of SCLC enhances our comprehension of the molecular mechanisms underlying this aggressive malignancy and offers innovative strategies for more effective clinical interventions.

Furthermore, methods based on bioinformatics and omics analysis have played a critical role in the research and development of targeted drugs for a variety of cancers such as breast cancer (Neagu et al., 2023), triple-negative breast cancer (TNBC) (Kudelova et al., 2022), gastric cancer (Hou et al., 2023), lung cancer (Yan et al., 2024; Rosenquist et al., 2023), and hematological malignants, greatly accelerating the discovery of new drug targets. In 2022, Alam et al. (2022) identified seven differentially expressed core genes while studying the molecular mechanisms of breast cancer progression. Through further multivariate survival analysis and regulatory network analysis, they proposed three reusable drugs guided by KGS (tramitinib, serumetinib, and RDEC119) for breast cancer treatment, and verified the effective binding ability of these drugs through molecular docking analysis. The genomic instability and high mutation rate of TNBC may lead to the production of neoantigens, thereby enhancing its immunogenicity, which poses great challenges for treatment. Current research focuses on the combination of immune checkpoint inhibitors with chemotherapy, PARP inhibitors, cancer vaccines, or natural killer cell therapies. In recent years, significant progress has been made in clinical studies of TNBC treatment, and based on these results, several effective drugs have been approved to benefit TNBC patients. These include the PARP inhibitors olaparib and talazoparib for the treatment of germ-line BRCA gene mutation-associated breast cancer (gBRCAm-BC), and immunotherapy for advanced TNBC with programmed cell death ligand-1 positive (PD-L1+), Combined use of the checkpoint inhibitor atezolizumab with albumin-binding paclitaxel (Shen et al., 2020). In addition, immunotherapies, which reshape the host immune system to destroy tumor cells, may lead to new treatment strategies. In immunotherapy for gastric cancer, accurate identification of predictive biomarkers holds promise for optimizing patient selection and improving treatment outcomes. With the advancement of bioinformatics technology, more biomarkers have been discovered and applied in immunotherapy for gastric cancer, such as PD-L1, MSI-H, dMMR, tumor mutation load (TMB) and Epstein-Barr virus (EBV) (Hou et al., 2023). In 2017, the U.S. Food and Drug Administration approved Bologlizumab for the treatment of unresectable or metastatic solid tumors in the MSI-H/dMMR state (Dudley et al., 2016). By integrating multi-omics techniques and establishing a progressive analysis framework from genetic basis to environmental exposure, the pathogenesis, pathophysiological process and molecular basis of immunotherapy of lung cancer can be deeply analyzed, thus providing strong support for immunotherapy of lung cancer (Yan et al., 2024). In the precision treatment of hematological malignancies, the combination of *in vitro* drug screening and multi-omics technology can provide a new treatment option for advanced patients (Rosenquist et al., 2023). Guo et al. (2024) revealed the mechanism of TiaoPi AnChang Decoction (TPACD) treating colorectal cancer by integrating UHPLC-Q-TOF-MS/MS, network pharmacology and bioinformatics techniques, and its anti-cancer effect was realized by targeting MMP3. It is reported that Esketamine can negatively regulate the proliferation and metastasis of cancer cells, and through further bioinformatics analysis, it was found that esketamine may show anti-esophageal squamous cell carcinoma properties by affecting the expression of ERCC6L, AHR and KIF2C proteins (Li et al., 2023). In summary, the rapid development of bioinformatics technology is of significant help to the development of new drugs and targets, as well as the clarification of the anti-cancer mechanism of existing drugs, and has greatly accelerated the development process of cancer targeted drugs.

### 3.2 The role of bioinformatics in drug research for epidemic diseases

Bioinformatics tools and multi-omics combined analysis have played an important role in responding to new disease outbreaks and developing new drugs. By analyzing the genomic information of pathogens, researchers can track the spread, evolution, and drug resistance of pathogens, which has important implications for controlling infectious diseases. Coronavirus disease (COVID-19), caused by severe acute respiratory syndrome Coronavirus 2 (SARS-CoV-2), has become a major global concern (Bala et al., 2021). At that time, drugs and vaccines were urgently needed to effectively combat the disease. Because of the huge challenges of new drug discovery, reusing existing drugs can reduce time and cost compared to developing new drugs from scratch. Haseeb Nisar et al. constructed a protein-protein interaction (PPI) network by analyzing differentially expressed genes (DEGs) in the RNA-seq transcripome dataset and integrating COVID-19 related genes from different databases (Nisar et al., 2024). Drug reuse analysis of the identified genes/proteins was conducted through the relevant information in the database, and finally the drug candidates (picetanol, CKD-712, and PMID26394986-Compound-10) were identified. Finally, by molecular docking analysis of drug-gene interactions, and verified by molecular dynamics simulation of 80 ns, PMID26394986-Compound-10 was identified as the only potential drug. But its effectiveness has yet to be assessed.

SARS-CoV-2 is highly contagious, so there is an urgent need to develop a vaccine for effective prevention. Since SARS-CoV-2 was first identified, scientists have rapidly analyzed its genome sequence using sequencing techniques (Wu et al., 2020). On this basis, Jin et al. (Jin et al., 2020) soon entered the first structure of SARS-CoV-2 into the protein database, and then the structure of SARS-CoV-2 protein was also broken through. With the help of bioinformatics technology, the structural study of SARS-CoV-2 has made remarkable progress, and numerous potential drugs and vaccines against SARS-CoV-2 have been developed (Wu et al., 2022). At that time, the 128 COVID-19 vaccines and vaccine candidates announced by the World Health Organization to enter clinical trials can be divided into three categories: first, protein vaccines, which produce target antigens *in vitro*, such as inactivated virus vaccines, virus-like particles and protein subunit vaccines; Second, genetic vaccines, such as viral vector vaccines, DNA vaccines and mRNA vaccines, deliver the genes encoding viral antigens into the host cells for *in vivo* production; A third class combines protein and gene approaches to produce protein antigens in and out of the body, typically represented by live attenuated vaccines. The RBD-dimer-based COVID-19 vaccine ZF2001 has shown excellent safety and efficacy in Phase 3 clinical trials, showing protection against symptomatic and severe COVID-19 for at least 6 months after complete vaccination (Dai et al., 2022). Other vaccines, such as BNT162b2, mRNA-1273, AZD1222, Ad26.COV2-S, Sputnik V, Covaxin, CoronaVac, BBIBP-CorV, and EpiVacCorona, have also shown good efficacy in Phase III trials. It has been approved for use in adults and, in some cases, in adolescents.

In addition, during the COVID-19 pandemic, bioinformatics techniques are also being used in drug development for co-existing diseases of other systems. Given the numerous overlapping clinical symptoms between COVID-19 and systemic lupus erythematosus (SLE), the potential existence of shared pathological mechanisms between the two remains an area for further investigation, particularly regarding the treatment of SLE patients infected with COVID-19. Wu et al. extracted common differentially expressed genes (DEGs) from datasets of both diseases and performed analyses on functional enrichment, pathway identification, and drug candidate screening. Their findings revealed that COVID-19 and SLE patients share several key hub genes, associated pathways, and regulatory networks (Wu et al., 2024). Building on these shared targets, they also identified a range of promising drug candidates for the treatment of patients with COVID-19, including those with concurrent SLE.

## 4 Conclusion and prospect

Bioinformatics tools and technologies in drug discovery include biological databases, molecular docking computing tools and omics techniques. In this paper, bioinformatics technology and its role and application in drug development are summarized in detail. Biological database is used to store, manage and share biological data, and collect existing research results and technical information for research workers to query and promote the development of new drugs. Molecular docking computing tools assist in determining the correct lead compound. Omics techniques such as genomics, transcriptomics, proteomics, and metabolomics play a significant role in drug research. By analyzing large amounts of genomic data, genomics can help researchers identify genes associated with diseases, develop targeted drugs, promote the development of personalized medicine, and formulate treatment plans based on an individual’s genomic information. Transcriptomics is used to analyze transcriptome data, the collection of all RNA in a given cell or tissue under a given condition, to help identify disease-associated changes in gene expression, potential biomarkers for disease diagnosis and treatment. By predicting the three-dimensional structure of proteins, proteomics provides clues to their role in disease and is critical to understanding protein function and drug design. Metabolomics involves the study of small molecules that are pivotal in identifying novel drug targets for cancer treatment. By integrating multi-omics data, bioinformatics also shows great potential in the treatment of cancer and epidemic diseases. In cancer therapy, various omics techniques enable a comprehensive study of the molecular characteristics of tumors and identify potential drug targets. By analyzing the genomic information of pathogens, multi-omics technology can track the spread, evolution and drug resistance of pathogens, which is of great significance for the control of infectious diseases.

Currently, we are experiencing a period of significant advances in drug research, thanks to technological advances, especially bioinformatic-based tools and methods. These advances have greatly enhanced our understanding of the biological roles and regulatory mechanisms of target molecules in disease. However, these advances also present significant challenges in accurately identifying target molecules in samples. How to accurately identify drug targets at a lower cost, and how to effectively use emerging artificial intelligence technologies to promote the identification and characterization of target molecules are urgent issues that need urgent attention.
